# Prevention of postoperative bleeding after complex pediatric cardiac surgery by early administration of fibrinogen, prothrombin complex and platelets: a prospective observational study

**DOI:** 10.1186/s12871-020-01217-1

**Published:** 2020-12-18

**Authors:** Nils Dennhardt, Robert Sümpelmann, Alexander Horke, Oliver Keil, Katja Nickel, Sebastian Heiderich, Dietmar Boethig, Christiane E. Beck

**Affiliations:** 1 Clinic for Anaesthesiology and Intensive Care Medicine, Hannover Medical School, OE 8050, Carl-Neuberg-Str. 1, D-30625 Hannover, Germany; 2 Clinic for Cardiac, Thoracic, Transplant and Vascular Surgery, Hannover Medical School, Carl-Neuberg-Str. 1, D-30625 Hannover, Germany

**Keywords:** Children, Bleeding, Cardiopulmonary bypass, Fibrinogen, Thrombelastography

## Abstract

**Background:**

Postoperative bleeding is a major problem in children undergoing complex pediatric cardiac surgery. The primary aim of this prospective observational study was to evaluate the effect of an institutional approach consisting of early preventive fibrinogen, prothrombin complex and platelets administration on coagulation parameters and postoperative bleeding in children. The secondary aim was to study the rate of re-intervention and postoperative transfusion, the occurrence of thrombosis, length of mechanical ventilation, ICU stay and mortality.

**Methods:**

In fifty children (age 0–6 years) with one or more predefined risk factors for bleeding after cardiopulmonary bypass (CPB), thrombelastography (TEG) and standard coagulation parameters were measured at baseline (T1), after CPB and reversal of heparin (T2), at sternal closure (T3) and after 12 h in the ICU (T4). Clinical bleeding was evaluated by the surgeon at T2 and T3 using a numeric rating scale (NRS, 0–10).

**Results:**

After CPB and early administration of fibrinogen, prothrombin complex and platelets, the clinical bleeding evaluation score decreased from a mean value of 6.2 ± 1.9 (NRS) at T2 to a mean value of 2.1 ± 0.8 at T3 (NRS; *P* <  0.001). Reaction time (R), kinetic time (K), maximum amplitude (MA) and maximum amplitude of fibrinogen (MA-fib) improved significantly (*P* <  0.001 for all), and MA-fib correlated significantly with the clinical bleeding evaluation (*r* = 0.70, *P* <  0.001). The administered total amount of fibrinogen (mg kg^− 1^) correlated significantly with weight (*r* = − 0.42, *P* = 0.002), priming volume as percentage of estimated blood volume (*r* = 0.30, *P* = 0.034), minimum CPB temperature (*r* = − 0.30, *P* = 0.033) and the change in clinical bleeding evaluation from T2 to T3 (*r* = 0.71, *P* <  0.001). The incidence of postoperative bleeding (> 10% of estimated blood volume) was 8%. No child required a surgical re-intervention, and no cases of thrombosis were observed. Hospital mortality was 0%.

**Conclusion:**

In this observational study of children with an increased risk of bleeding after CPB, an early preventive therapy with fibrinogen, prothrombin complex and platelets guided by clinical bleeding evaluation and TEG reduced bleeding and improved TEG and standard coagulation parameters significantly, with no occurrence of thrombosis or need for re-operation.

**Trial registration:**

German Clinical Trials Register DRKS00018109 (retrospectively registered 27th August 2019).

## Background

Postoperative bleeding is a major problem in children with congenital heart disease undergoing complex pediatric cardiac surgery with cardiopulmonary bypass (CPB) and results in significant postoperative morbidity and mortality [1, 2]. Risk factors associated with a higher incidence of bleeding are an age of less than 1 year, hypothermia on CPB, longer CBP duration and re-sternotomy [3]. For the prevention of massive bleeding, blood products and coagulation factor concentrates can be used early after CPB [4, 5]. In our institution, fibrinogen, prothrombin complex concentrate and platelets are used routinely in children with an increased bleeding risk until sufficient hemostasis is achieved and surgical closure of the thorax is possible. More than ten years of experience have shown this approach to be safe and effective, but no objective evaluation has been performed yet. Therefore, we conducted a prospective clinical observational study to investigate the impact of our institutional approach on children with increased risk of postoperative bleeding. The primary aim was to evaluate the effect on standard coagulation parameters, thrombelastography and postoperative bleeding. The secondary aim was to study the rate of re-intervention and postoperative transfusion, the occurrence of thrombosis, length of mechanical ventilation, ICU stay and mortality.

## Methods

### Patients

**Fig. 1:**
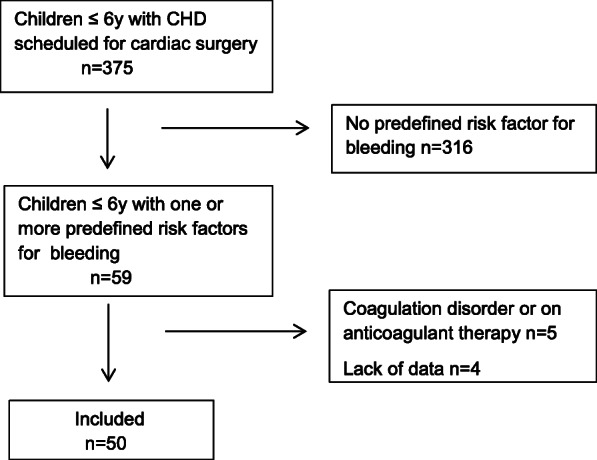
Study flow chart

This prospective observational study was conducted according to the standards set forth by the Declaration of Helsinki and Good Clinical Practice guidelines. Following the local ethics committee’s approval (Ethics Committee of Hanover Medical School, Germany, Chairperson Prof. Dr. H. D. Troeger, No. 7349 dated February 2, 2017), 50 children ranging from 0 to 6 years of age scheduled for cardiac surgery with CPB and one or more risk factors for bleeding were included (Fig. 1). The risk factors were predefined as an age of less than 1 year, hypothermia on CPB < 32 °C, expected CPB duration > 90 min, re-sternotomy or extensive aortic suture lines. Children with pre-existing coagulation disorders or on anticoagulant or antiplatelet therapy were excluded. The study was conducted from April 2017 to November 2018 at the Clinic for Anesthesiology and Intensive Care Medicine, Hanover Medical School, Germany, and all operations were performed by the same team of surgeons.

### Intraoperative management

Anesthesia was induced by injection of 0.5 mg kg^− 1^ etomidate, 0.5 μg kg^− 1^ sufentanil and 0.5 mg kg^− 1^ atracurium and maintained by sufentanil 1 μg kg^− 1^ h^− 1^ and sevoflurane (on CPB administered via oxygenator).

CPB was performed with the heart-lung machine LivaNova S5 (LivaNova PLC, London, UK) and the oxygenator system TerumoFX05 (Terumo Corporation, Tokyo, Japan). The system was prepared in a standardized fashion: The circuit was primed with a bicarbonate-buffered hemofiltration solution (BB-HS; Duosol, B. Braun, Melsungen, Germany), 2 mL kg^− 1^ mannitol, 150 IU kg^− 1^ of heparin and 20 mL kg^− 1^ gelatin. In infants with a body weight below 5 kg, 10 mL kg^− 1^ albumin 20% was used instead of gelatin. Packed red blood cells were added if necessary to achieve hemoglobin levels of 8–10 g dL^− 1^. According to patient’s weight, the total priming volume fluctuated between 180 and 450 ml. To achieve a physiological composition, priming volume was hemofiltered before CPB using a polysulfone hemofilter (ME HF0S 0020, Medos AG, Stolberg, Germany) by ten minutes circulation until approximately 1000 mL of ultrafiltrate were restored by BB-HS [6]. During the last 30 min of CPB, 20–30 mL kg^− 1^ fresh frozen plasma was added, and a higher amount of fluid was removed by hemofiltration. Target pump flow was 2.7 L min^− 1^ m^− 2^ for children and 3.0 L min^− 1^ m^− 2^ for infants below one year of age. Target mean arterial pressure was guided by near-infrared spectroscopy (NIRS) and continuously measured central venous oxygen saturation in the venous line of the bypass. Before CPB, a heparin bolus of 400 IU kg^− 1^ was given to achieve anticoagulation. During CPB, activated clotting time (ACT) was maintained longer than 400 s by adding additional heparin if necessary. Tranexamic acid was administered at 10 mg kg^− 1^ h^− 1^. At the end of CPB, the administered heparin was reversed by protamine starting at a ratio of 0.8 until the ACT had returned to < 130 s.

After CPB weaning, heparin reversal and clinical bleeding evaluation, the hemostatic therapy was started with 50 mg kg^− 1^ human fibrinogen (Haemocomplettan®, CSL Behring GmbH, Marburg, Germany), 50 IU kg^− 1^ human prothrombin complex (Beriplex®, CSL Behring GmbH, Marburg, Germany) and 20 mL kg^− 1^ platelets. Repeat doses were guided by clinical bleeding evaluation and TEG as follows: In case of MA-fib < 15 mm, fibrinogen was added; in case of R > 9.5 min, prothrombin complex was added; and in case of MA < 52 mm and normal MA-fib (> 15 mm), platelets were added until the bleeding situation improved clinically significant and closure of the thorax was possible.

### Data collection

Results of blood gas analysis, hematologic (hemoglobin, hematocrit, platelets), coagulation standard (Quick value of prothrombin time (Quick), activated partial thromboplastin time (aPTT), activated clotting time (ACT), fibrinogen (Clauss method), antithrombin III, factor II, factor V and TEG parameters (reaction time (R), kinetic time (K), angle, maximum amplitude (MA), functional fibrinogen, maximum amplitude of functional fibrinogen (MA-fib), fibrinolysis at 30 min after maximum amplitude (LY30)) were collected at the following points in time: at baseline before skin incision (T1); after CPB and reversal of heparin by protamine before administration of coagulation factors or blood products (T2); at sternal closure (T3); and after 12 h in the ICU (T4). For the TEG analysis, the TEG 6 s analyzer (Haemonetics, Braintree, Massachusetts, USA) was used. At T2 and T3, the operating surgeon was asked to evaluate the bleeding on a numeric rating scale from 0 to 10 (NRS; 0 = absolutely dry, no signs of any bleeding at all; 10 = massive bleeding with no signs of coagulation). The administered coagulation factors and blood products were documented. Intraoperative data included CPB time, cross-clamp time, duration of deep hypothermic circulatory arrest (if used) and the lowest temperature during CPB. Postoperative data included chest drainage output within the first six postoperative hours, rate of re-intervention and postoperative transfusion, the occurrence of thrombosis, length of mechanical ventilation, ICU stay and mortality. Significant postoperative bleeding was defined as a blood loss of more than 10% of the child’s estimated blood volume within the first six postoperative hours.

### Statistics

Data were recorded in an Excel database, analyzed using MS Excel (Excel 2010; Microsoft, Seattle, USA) and GraphPad Prism (Prism 7; Graph Pad Software Inc., San Diego, USA) software tools, and presented as mean values plus standard deviation (range) or as median (range). Spearman correlation, regression analysis and independent-samples Mann-Whitney-U tests were performed with a pre-defined significance level of α = 0.05.

## Results

A total of 50 children were included. Surgical procedures are summarized in Table 1. Demographic, intra- and postoperative data are summarized in Table 2.

**Table 1:** Surgical procedure type (*n* = 50)

Procedures	n
TOF repair	7
AA repair	6
ASO	5
Fontan	5
ASO + AA repair	3
TAC repair	3
TE-AV replacement	3
bilateral Glenn	3
ASO + VSD repair	2
TAPVC repair	2
CAVC repair	2
AV repair	2
AA repair + VSD repair	1
Rastelli	1
other	5

*Abbreviations*: *TOF* tetralogy of Fallot, *AA* aortic arch, *ASO* arterial switch operation, *VSD* ventricular septal defect, *TAC* truncus arteriosus communis, *TE-AV* tissue-engineered aortic valve, *TAPVC* total anomalous pulmonal vein connection, *CAVC* complete atrio-ventricular channel, *AV* aortic valve

**Table 2:** Demographic, intra-, and postoperative data (*n* = 50). Data are presented as mean ± standard deviation (range) or number (percentage)

Age (months)	15.6 ± 22.5 (0–74)
Weight (kg)	8.1 ± 5.9 (2.0–24.2)
Height (cm)	70.1 ± 21.2 (47–118)
Cyanotic (n)	32 (64%)
Neonate (n)	12 (24%)
Re-operation (n)	19 (38%)
Hypothermia < 32 °C (n)	42 (84%)
Priming Volume (% of EBV)	57 ± 24 (25–112)
Lowest temp.(°C)	24.9 ± 4.8 (17.9–34.0)
CPB time (min)	231 ± 84 (91–492)
Cross-clamp time (min)	127 ± 62 (28–256)
DHCA (min)	24 ± 6 (8–32) (*n* = 11)
Drainage volume within 6 h (% of EBV)	6.7 ± 3.7 (2.3–20.2)
Drainage volume within 6 h > 10% of EBV (n)	4 (8%)
Surgical re-intervention (n)	0
Thrombosis (n)	0
Mechanical ventilation duration (h)	39 ± 51 (2–255)
ICU stay (days)	5.0 ± 3.9 (1–19)
Mortality (%)	0

*Abbreviations*: *EBV* estimated blood volume, *CPB* cardiopulmonary bypass, *DHCA* deep hypothermic circulatory arrest, *ICU* intensive care unit

Besides hemoglobin concentration, there were no significant differences between cyanotic and non-cyanotic children in measured hematologic, coagulation standard or TEG parameters at baseline (T1). After CPB and administration of protamine (T2), hemoglobin, hematocrit, platelets, Quick, fibrinogen, AT III, factor II, angle, MA, MA-fib and LY30 were significantly reduced, and R and K were significantly extended, as compared to the baseline values (T1) (*P* <  0.001 for all). ACT, aPTT and factor V did not differ significantly (Table 3). At this point in time (T2), the surgeon evaluated clinical bleeding at a mean value of 6.2 ± 1.9 on a numeric rating scale, and the administration of fibrinogen (mean total amount 108 ± 56 mg kg^− 1^), prothrombin complex concentrate (mean total amount 70 ± 26 IU kg^− 1^), platelets (mean total amount 22 ± 14 mL kg^− 1^) and red blood cells (mean total amount 18.9 ± 18 mL kg^− 1^) was started. In combination with surgical hemostasis, the results of the clinical bleeding evaluation score reduced significantly to a mean of 2.1 ± 0.8 (*P* <  0.001). At sternal closure (T3), platelets, Quick, fibrinogen, factor II, angle, MA and MA-fib had significantly increased, and R and K had significantly decreased (*P* <  0.001 for all, Table 3).

**Table 3:** Hematologic, coagulation standard and TEG parameters, and clinical bleeding as assessed by surgeon at baseline (T1), after bypass and administration of protamine (T2), at sternal closure (T3), and in ICU after 12 h (T4). Data are presented as median (range)

	T1	T2	T3	T4	*P* (T1 vs T2)	95%-CI for *P* (T1 vs T2)	*P* (T2 vs T3)	95%-CI for *P* (T2 vs T3)
Hb (g dl^−1^)	13.4 (8.2–18.4)	11.4 (8.5–15.8)	12.2 (9.3–16.9)	13.7 (9.5–17.8)	< 0.001	1.4 to 3.1	ns	
Hct (%)	40.6 (25.2–55.1)	34.9 (24.1–48.5)	36.7 (28.5–49.0)	38.1 (26.9–52.5)	< 0.001	− 23.7 to 0.51	ns	
PLT (10^9^ L^−1^)	279 (155–621)	116 (68–266)	219 (112–329)	246 (123–380)	< 0.001	− 233 to − 169.5	< 0.001	83.9 to 121.
Quick (%)	92 (54–129)	49 (40–82)	85 (62–130)	83 (59–130)	< 0.001	− 44.1 to −33.8	< 0.001	28.6 to 37.9
aPTT (s)	32 (23–50)	41 (31–55)	38 (26–59)	31 (24–49)	ns		ns	
ACT (s)	110 (91–148)	114 (91–146)	106 (79–138)	110 (89–126)	ns		ns	
Fib (g L^−1^)	1.7 (0.9–3.3)	1.4 (0.3–2.2)	2.2 (1.5–3.5)	2.5 (1.3–3.8)	< 0.001	−0.49 to − 0.14	< 0.001	0.6 to 1.0
AT III (%)	89 (44–120)	67 (47–97)	67 (47–91)	76 (46–101)	< 0.001	−28.1 to − 14.3	ns	
F II (%)	60 (32–94)	49 (34–87)	113 (70–170)	101 (62–167)	0.04	−12.5 to − 1.5	< 0.001	56.3 to 72.2
F V (%)	77 (57–121)	61 (37–81)	60 (32–89)	72 (33–148)	ns		ns	
R (min)	6.9 (4.3–10.7)	10.0 (5.7–15.3)	7.9 (5.2–12.1)	7.2 (3.5–11.2)	< 0.001	1.9 to 3.1	< 0.001	−2.2 to −0.6
K (min)	1.5 (0.9–4.3)	2.8 (0.9–7.4)	1.8 (1.0–3.7)	1.2 (0.7–2.1)	< 0.001	0.7 to 1.5	< 0.001	−1.4 to −0.6
Angle (degree)	70.7 (48.4–77.8)	59.1 (38.0–77.4)	68.9 (56.9–77.8)	74.4 (63.9–81.7)	< 0.001	− 11.7 to −5.9	< 0.001	5.9 to 11.3
MA (mm)	59.0 (43.9–69.7)	48.9 (30.5–64.1)	62.0 (50.6–68.6)	64.3 (55–69.6)	< 0.001	−12.0 to – 7.3	< 0.001	9.1 to 13.5
MA-fib (mm)	19.6 (11.1–35.5)	13.2 (4.9–21.0)	29.1 (19.5–53.2)	32.0 (23.0–50.6)	< 0.001	−9.5 to −6.1	< 0.001	15.5 to 19.9
LY30 (%)	0.6 (0–5.1)	0.0 (0–3.2)	0.0 (0–3.8)	0.6 (0–2.7)	0.008	−1.1 to 0.2	ns	
Bleeding (NRS 0–10)		6 (2–10)	2 (0–4)				< 0.001	−4.8 to −3.6

*Abbreviations*: *ns* not significant, *Hb* haemoglobin, *Hct* haematocrit, *PLT* platelets, *Quick* Quick value of prothrombin time, *aPTT* activated partial thromboplastin time, *ACT* activated clotting time, *Fib* fibrinogen, *AT III* Antithrombin, *F II* factor II, *F V* factor V, *R* reaction time, *K* kinetic time, *MA* maximum amplitude, *MA-fib* maximum amplitude of functional fibrinogen, *LY30* fibrinolysis at 30 min

**Fig. 2:**
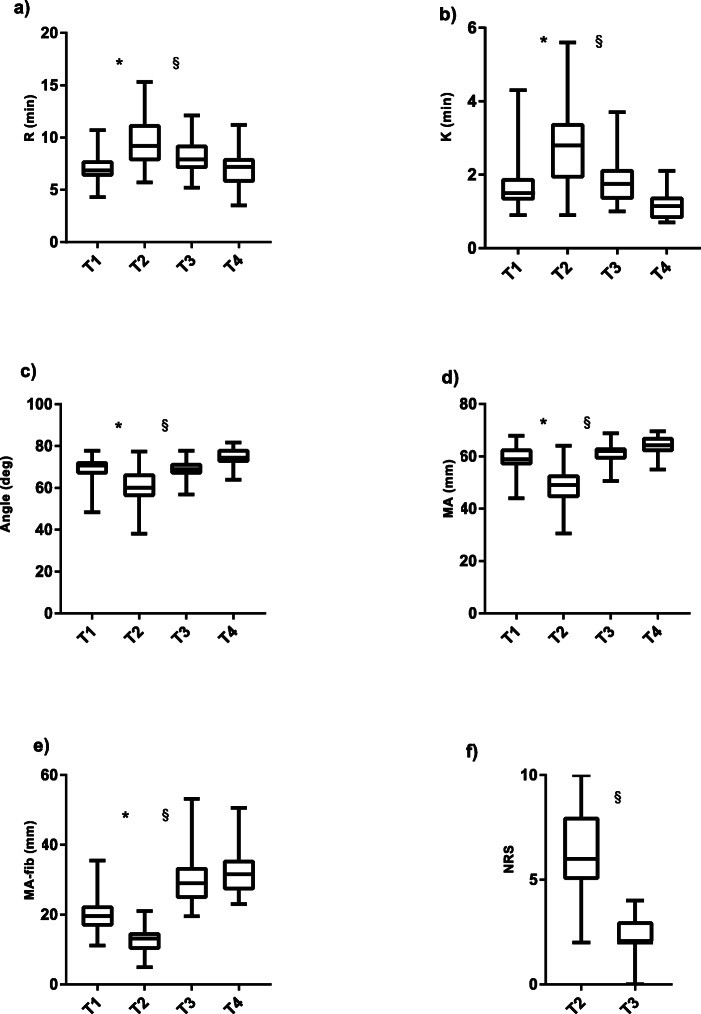
Box and whisker plots (95th, 75th, 50th, and 5th percentiles) of TEG parameters (**a-e**) and clinical bleeding evaluated by surgeon (**f**) at baseline (T1), after bypass and administration of protamine (T2), at sternal closure (T3), and in ICU after 12 h (T4). Abbreviations: R, reaction time; K, kinetic time; MA, maximum amplitude; MA-fib, maximum amplitude of functional fibrinogen; NRS, numeric rating scale; * *P* < 0.001 (T1 vs T2), § *P* < 0.001 (T2 vs T3)

**Fig. 3:**
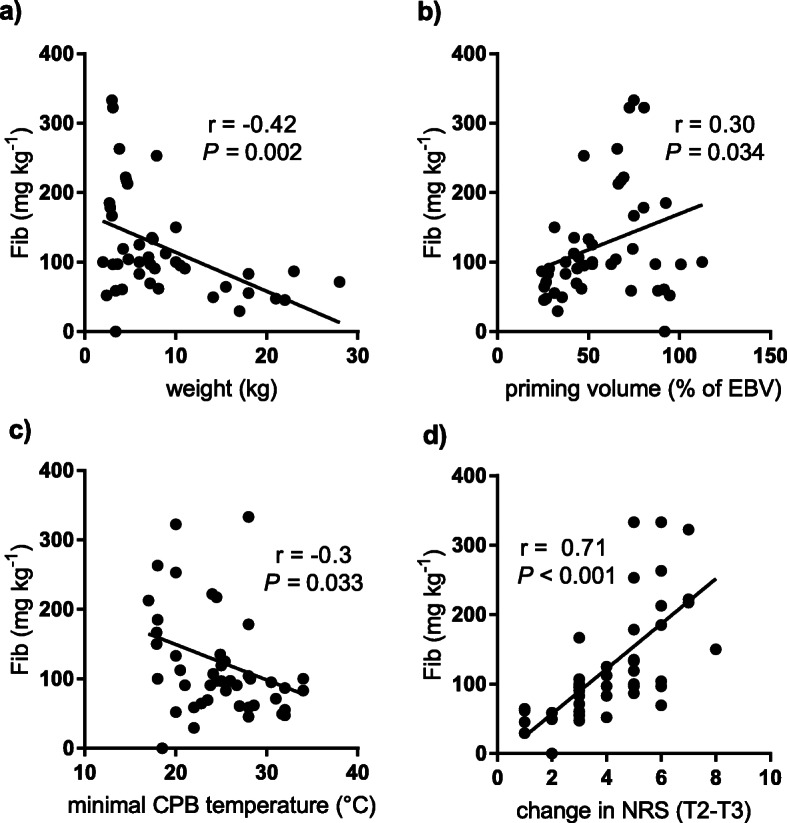
Correlation between administered total amount of fibrinogen kg^− 1^ and weight (**a**), priming volume as percentage of estimated blood volume weight (**b**), minimum temperature during cardiopulmonary bypass (**c**) and change in clinical bleeding evaluation from T2 to T3 (**d**). Abbreviations: Fib, fibrinogen; EBV, estimated blood volume; CPB, cardiopulmonary bypass; NRS, numeric rating scale

Results of TEG parameters and clinical bleeding evaluation are shown in Fig. 2. MA-fib correlated significantly with the clinical bleeding evaluation (*r* = 0.70, *P* < 0.001). The administered total amount of fibrinogen (mg kg^− 1^) correlated significantly with age (*r* = − 0.41, *P* = 0.003), weight (*r* = − 0.42, *P* = 0.002), priming volume as percentage of estimated blood volume (*r* = 0.30, *P* = 0.034), minimum CPB temperature (*r* = − 0.30, *P* = 0.033) and the change in clinical bleeding evaluation from T2 to T3 (*r* = 0.71, *P* < 0.001; Fig. 3). Patients weighing less than 8 kg had significantly higher scores in clinical bleeding evaluation after CPB (*P* = 0.039) and received significantly higher fibrinogen doses thereafter (mg kg^− 1^, *P* = 0.002). The plasma fibrinogen levels (Clauss method) correlated significantly with functional fibrinogen (*r* = 0.76) and MA-fib (*r* = 0.75, *P* < 0.0001 for both). The results of systemic perfusion after CPB evaluated by arteriovenous oxygen saturation difference (mean 26.8 ± 8.9) were adequate in all cases.

In the ICU (T4), hematologic, coagulation standard and TEG parameters after 12 h remained stable. The mean chest drainage volume within the first six postoperative hours was 5.3 ± 2.9 (1.8–16.1) mLkg^− 1^; this is equivalent to 6.7 ± 3.7 (2.3–20.2) % loss of EBV. Four children (8%) had a postoperative blood loss of > 10% of EBV. Nine children (18%) received further red blood cell transfusions (mean 11.4 ± 2.4, 8–16 mL kg^− 1^) and one child 50 mg kg^− 1^ fibrinogen. Length of mechanical ventilation was 39 ± 51 (2–255) hours, and the mean ICU stay was 5.0 ± 3.9 (1-19) days. No child needed surgical re-intervention, no case of thrombosis was observed, and overall mortality was 0%.

## Discussion

Our study showed that nearly all children with risk factors for bleeding presented with significant coagulation disorders after CPB, that an early preventive therapy with fibrinogen, prothrombin complex concentrate and platelets improved the hemostasis with no occurrence of thrombosis or need for re-operation and that a low body weight and a low CPB temperature were associated with higher administered fibrinogen doses (mg kg^− 1^).

Excessive bleeding after cardiopulmonary bypass is a serious complication and may be caused by coagulopathy and insufficient surgical hemostasis. Contributory mechanisms include heparinization and hemodilution through the circuit prime, exposure of blood to artificial surfaces, hypothermia and interactions between the inflammatory and coagulation systems [7]. Maintenance of a stable homeostasis is of high importance and therefore bicarbonate- buffered ultrafiltration was favored to achieve a physiologic priming solution and to prevent electrolyte and acid- base disturbances [6, 8]. Fresh frozen plasma (FFP) was added during rewarming and ultrafiltration on CPB to avoid fluid overload, but the results of clinical bleeding evaluation, TEG and standard coagulation parameters at T2 showed that FFP alone was not effective to prevent bleeding. Therefore, coagulation factor concentrates and platelets were added subsequently, and this strategy improved the results of clinical bleeding evaluation and thrombelastography significantly.

Prothrombin complex concentrates contain coagulation factors required to directly promote thrombin generation [5]. Studies including adults after CPB suggest that the use of prothrombin complex concentrate as part of a multimodal coagulation management strategy may have blood sparing effects [9, 10], but there is a lack of randomized controlled studies in both adult and pediatric patients. The main concern regarding the use of prothrombin complex concentrate is the possibility of thrombotic complications especially in children with a low cardiac output and artificial shunts [11]. Neither in our study or in our clinical practice have cases of thrombosis been observed, but the sample size was too low to assess safety. Therefore, the individual risk of thrombosis should be weighed against the perioperative risk of bleeding when using prothrombin complex concentrates in children undergoing complex pediatric cardiac surgery.

Fibrinogen is the major substrate for clot formation and additionally promotes platelet activation and agglomeration. Hemodilution from CPB results in decreased fibrinogen levels, which in turn results in impaired fibrin formation, inadequate clot formation and bleeding. Faraoni et al. [12] demonstrated in a retrospective analysis that the post-CPB plasma fibrinogen concentration significantly impacts blood loss in children undergoing cardiac surgery. Downey et al. [13] suggested that fibrinogen concentrate may be considered as an alternative to cryoprecipitate in infants with bleeding after CPB, but cryoprecipitate is not available in Germany. Mahovec et al. demonstrated that measuring fibrinogen levels during the rewarming phase of CPB reduced cryoprecipitate transfusion [14]. An animal experimental study showed that hemotherapy with fibrinogen did not affect arterial thrombogenesis [15]. Guidelines by the European Society of Anesthesiology recommend to keep the level of plasma fibrinogen at no less than 1.5 to 2 g L^− 1^ in bleeding patients [16]. In our study, most children had lower levels immediately after CPB but all had higher levels after fibrinogen administration at sternal closure. Therefore, keeping fibrinogen levels within a normal range should be one of the main goals to prevent bleeding in children undergoing complex cardiac surgery.

Platelet count and aggregation are markedly reduced after CPB, especially in neonates, and patients with impaired platelet function during CPB had markedly increased intraoperative transfusion requirements [17, 18]. Activation and aggregation of platelets following blood contact with foreign material, systemic inflammation, adverse effects of heparin on platelets and fibrinolytic system and hypothermia are among the most significant factors, but the relationship between postoperative bleeding and platelets dysfunction is a subject that is still being debated [4]. In our study, platelet counts were decreased in many children after CPB, but within a normal range after platelet transfusion at sternal closure. The platelet function was not analyzed, and therefore the hemostatic effect of the platelets transfused is unclear.

The TEG analyzer used in this study requires only a 300-μL blood sample and processes the sample automatically without a time-consuming pipette procedure. The results of the functional fibrinogen measurements correlated with the Clauss method, which is in accordance with a previous study by Gautam et al. [19]. To avoid a time delay, the hemostatic therapy should be started early after CPB if diffuse bleeding is clinically evident. Thereafter, the results of TEG analysis can be used to guide the hemostatic therapy in more detail until a stable clot formation is achieved [20]. In cases with difficult bleeding localization, this strategy can help to identify areas with surgical bleeding. Besides the results of TEG analysis, a close cooperation with the attending surgeon is important to evaluate the bleeding situation and the efficacy of the administered hemostatic therapy. Therefore, careful clinical evaluation should be performed and TEG analysis should be repeated to control success and to adapt the hemostatic therapy, if necessary. Cui et al. demonstrated that the use of TEG in children undergoing complex cardiac surgery reduced perioperative transfusion and improved the outcome [21].

In our study, neonates and infants < 8 kg had significantly higher scores in clinical bleeding evaluation after CPB and needed significantly higher fibrinogen doses (mg kg^− 1^) afterwards. These patients are at particular risk due to their immature coagulation systems and the mismatch between the CPB priming volume and the infants’ blood volume [7]. Therefore, minimizing the priming volume and circuit should be an important goal, especially for small infants, and several studies reported safe use of miniaturized CPB circuits with a significant reduction of blood product transfusions [22–24]. While the benefits should be weighed against the downsides (e.g., reduced access to patient and circuit, vacuum assisted drainage - with the risk of air entrainment and hemolysis – often required, need for a very well-trained perfusion team), these techniques will probably be increasingly used in the future.

The incidence of clinically significant postoperative bleeding (8%) defined as postoperative drainage volume > 10% of EBV within the first six postoperative hours was more than two times lower when compared to other studies (incidence > 20%) [25, 26], even though these studies included also children without risk factors for bleeding. The major difference in hemostatic approach was the use of coagulation factors (fibrinogen, prothrombin complex concentrate) in the presented study. Also, other factors than hemostatic management alone may have contributed to the different results.

The presented study does, however, have some limitations. We performed a single-center prospective observational study on a special patient group (children below six years of age with one or more risk factors for bleeding) and the results may not be applicable to other populations. The attending anesthesiologists and surgeons were not blinded, and the sample size was too low to assess safety. The study design was observational and a randomization into groups of different fixed coagulation therapy regimens or a control group was not possible because of ethical reasons.

In conclusion, in this observational study of children with an increased risk of bleeding after complex cardiac surgery, an early preventive therapy with fibrinogen, prothrombin complex and platelets guided by clinical bleeding evaluation and thromboelastography (TEG) reduced bleeding and improved TEG and standard coagulation parameters significantly, with no occurrence of thrombosis or need for re-operation. Further studies are necessary to assess safety.

## Data Availability

The datasets used and/or analyzed during the study are available from the corresponding author on reasonable request.

## Abbreviations

CPB: Cardiopulmonary bypass
NRS: Numeric rating scale
TEG: Thrombelastography
ACT: Activated clotting time
R: Reaction time
K: Kinetic time
MA: Maximum amplitude
MA-fib: Maximum amplitude of functional fibrinogen
LY30: Fibrinolysis at 30 min after maximum amplitude
ICU: Intensive care unit
FFP: Fresh frozen plasma
EBV: Estimated blood volume
